# Multi-dimensional quantitative model of communication network value

**DOI:** 10.1038/s41598-022-12501-0

**Published:** 2022-05-19

**Authors:** Lijuan Wu, Chaoqin Gan, Zhongsen Xu, Jianqiang Hui

**Affiliations:** Key Laboratory of Specialty Fiber Optics and Optical Access Networks, Joint International Research Laboratory of Specialty Fiber Optics and Advanced Communication, Shanghai University, Shanghai, 200444 China

**Keywords:** Electrical and electronic engineering, Fibre optics and optical communications, Computer science, Information technology

## Abstract

In this paper, a multi-dimensional quantitative model is firstly founded for evaluating communication network value, and the quantification of the abstract concept of network value is realized. By analyzing various factors that influence the evaluation of network value from multiple perspectives, an index system of multi-dimensional network value evaluation is established. By adopting the analytic hierarchy process (AHP) method, not only the weight of each dimension is reasonably determined, but also the weight of different indicators in the second dimension is determined. By the characteristics of each dimension and its weight in the evaluation system, a multi-dimensional quantitative model for evaluating communication network value is constructed. By analyzing the value of Ethernet passive optical network (EPON), the rationality of the proposed quantitative model is demonstrated.

## Introduction

As the important infrastructure of modern information society, communication network has a huge scale. And with the development of the times, the scale will expand rapidly^1,2^. How to make full use of huge network resources to serve the society better and more efficiently has attracted people (especially operators) to attach great importance^3–5^. The evaluation of communication network value provides key support for solving the above problems. By evaluating the value of communication networks, operators can precisely optimize their networks and rationally allocate resources. Therefore, the research on network value evaluation is of great significance and practical application value.

At present, the research on network value mainly focuses on the business model^6–8^, and the research on communication network value is still in the initial stage. Bob Metcalfe who first proposed the concept of network value only considered the number of network devices when defining network value^9^. Xia et al. defined the network value as the proportion of the priority of each service to the sum of the priorities of all services in virtual passive optical network (VPON), which is from a single perspective of service priority^10^. In^11^ and^12^, in addition to service priorities, network service value is also related to bandwidth. These studies only provide a simple definition of network value, without further research and analysis. Here, this paper will deeply analyze various factors that affect the evaluation of network value from multiple perspectives, and establish a quantitative model of communication network value. It will provide a theoretical basis for the evaluation and optimization of the network value in the future.

In this paper, we first propose a network value evaluation index system. In this multi-dimensional system, the evaluation indicators of network value are analyzed in detail from three dimensions, namely, safe and reliable value, QoS value and economic value. Then, a multi-dimensional network value quantification model is established according to the network value evaluation index system. The analytic hierarchy process is used to determine the weights of different dimensions and the weights of indicators in QoS value. It realizes the transformation of the evaluation of network value from qualitative description to quantitative calculation. Finally, a detailed network value analysis of Ethernet passive optical networks is performed.

The rest of this article is organized as follows. In section “The index system of multi-dimensional network value evaluation”, an index system of multi-dimensional network value evaluation is proposed. In section “Multi-dimensional quantitative model of network value”, the multi-dimensional quantitative model of network value is constructed. In section “Network value analyses”, the network value of Ethernet passive optical network (EPON) is calculated and analyzed. Finally, we conclude the paper in section “Conclusion”.

## The index system of multi-dimensional network value evaluation

**Figure 1:**
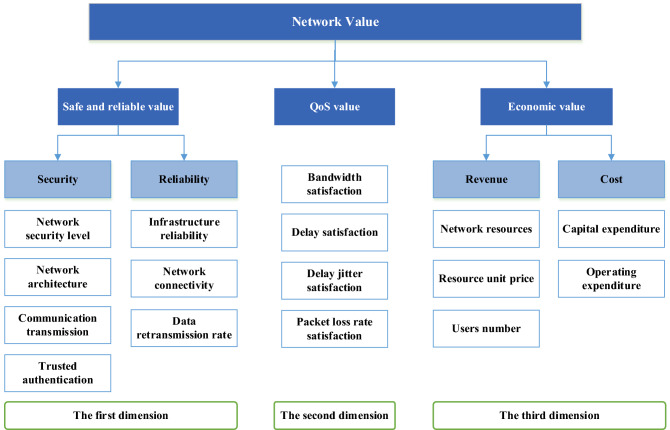
Network value evaluation index system.

For a communication network, the value of the network not only refers to the profit that the network can bring, but also refers to the quality of the network. Therefore, the evaluation of network value should be multi-dimensional. The network value evaluation index system is shown in Fig. 1. It can be seen from the figure that this paper constructs network value model from three dimensions. The first dimension is the safe and reliable value of network. The security and reliability of the network are taken as evaluation indexes to ensure that network can communicate successfully. The second dimension is the quality of service (QoS) value of network. Evaluating the value of a network in terms of its quality of service can reflect the performance of the network. The third dimension is the economic value of network. Network revenue as one of the metrics to measure network value can ensure that operators get higher profits from the network. The evaluation indicators of these three dimensions are described in detail below.

The safe and reliable value is regarded as the first-dimension value. This is because security and reliability are the most important performances for communication networks, and other performances of the network are based on this performance. The evaluation of this dimension’s value can be carried out from security and reliability respectively. Here, the security of network refers to the ability of network to resist external attacks. We all know that different networks are of different importance to national security, economic development and social life. Therefore, the requirements of security for different networks are different. The purpose of using network security level as one of the evaluation factors of network security value is to analyze different levels of networks differently so as to achieve more accurate value evaluation. The security index of network architecture mainly measures three aspects. One is to determine whether the network is divided into different network areas based on factors such as importance, and whether technical isolation measures are taken between important network areas and other network areas. It ensures the security of important network areas. The second is to determine whether the network provides hardware redundancy of communication lines, critical network equipment and critical computing equipment. This ensures the availability of the network system. The third is to determine whether the service processing capability of the network equipment and the bandwidth of each part of the network can meet the needs of service peak. This ensures the security of peak service. The indicator for communication transmission is to determine whether authentication techniques and security measures are used during data transmission. This indicator guarantees the integrity and confidentiality of the data during communication. Trusted authentication is to determine whether the network is based on a trusted root, to verify the system bootloader, system programs, important configuration parameters and communication applications of the communication device. And whether to alarm when the credibility of the device is destroyed. This index ensures that the network is not damaged by application software and viruses, and can effectively protect the security of the network system.

The reliability of the communication network here refers to the ability of network to successfully transmit data. It mainly includes three aspects: infrastructure reliability, network connectivity and data retransmission rate. Infrastructure reliability refers to the reliability of network hardware facilities. This index can be calculated according to the unreliability of network equipment in the design of network architecture. Generally, it must meet the condition of greater than 99.999%. Network connectivity refers to the ratio of the number of interconnected node pairs to the maximum possible number of node pairs in the network. It judges network reliability from overall connection of network. The larger the value of this indicator, the more paths the data has from the origin to the destination. Accordingly, the higher the probability of successful data transfer. Data retransmission rate refers to the ratio of the amount of data that needs to be retransmitted in the network to the total amount of data transmitted in the network. It judges the reliability of network from the data link transmission level. The larger the value, the less reliable the data link transmission.

QoS value, as the second dimension of network value, evaluates network value from the level of network service quality. ITU-T defines network inherent QoS as network performance, which can be reflected by parameters such as bandwidth, packet loss rate, delay and delay jitter. In order to describe the network quality of service more comprehensively, user perception is also taken into account in this paper. The QoS value of network can be reflected by users’ satisfaction with network. Therefore, the evaluation indexes of QoS value are bandwidth satisfaction, packet loss satisfaction, delay satisfaction and delay jitter satisfaction. Here, we introduce the user satisfaction model to represent these indicators. Referring to^13,14^, these four indicators are defined in the form of sigmoid functions, as shown in Eqs. (1)–(4). ${Q}_{bs}$, ${Q}_{pl}$, ${Q}_{d}$ and ${Q}_{j}$ represent bandwidth satisfaction, packet loss satisfaction, delay satisfaction and delay jitter satisfaction, respectively. ${b}_{m}$, ${d}_{m}$ and ${d}_{jm}$ represent user’s requested bandwidth, satisfied delay and satisfied delay jitter. $b$, $l$, $d$ and ${d}_{j}$ represent allocated bandwidth, packet loss rate, delay and delay jitter. $\delta$ is a parameter that controls the shape of the curve. The larger $\delta$ is, the more sigmoid function tends to be a step function.

1 $${Q}_{bs}=\frac{2}{1+{e}^{\delta ({b}_{m}-b)}}$$

2 $${Q}_{pl}=\frac{2}{1+{e}^{\delta l}}$$

3 $${Q}_{d}=\frac{1+{e}^{-\delta {d}_{m}}}{1+{e}^{-\delta ({d}_{m}-d)}}$$

4 $${Q}_{j}=\frac{1+{e}^{-\delta {d}_{jm}}}{1+{e}^{-\delta ({d}_{jm}-{d}_{j})}}$$

The last dimension is the economic value dimension. This dimension refers to the economic profit that the network can obtain. It can be calculated by revenue and cost of network. Network revenue refers to the total economic revenue obtained by selling network resources to users. It is mainly related to the total available resources of the network, the unit price of resources and the number of network users. Network costs refer to capital expenditures (CAPEX) and operational expenditures (OPEX). Capital expenditures refer to the initial set up cost of the network, mainly for network construction cost, including the cost of deploying network and network equipment cost^15^. Operational expenditures mainly refer to the cost of network operation and maintenance.

## Multi-dimensional quantitative model of network value

Section “The index system of multi-dimensional network value evaluation” analyzes the evaluation index system of network value. In this section, a multi-dimensional quantitative model of network value is constructed according to the evaluation index system.

### Quantitative model of safe and reliable value

The network security value is closely related to the security level requirements of network. Therefore, it is necessary to understand how many security levels a communication network has and the difference in requirements for each level. According to Baseline for Classified Protection of Cybersecurity^16^ and Evaluation Requirement for Classified Protection of Cybersecurity^17^, the network security protection level can be divided into five levels as shown in Table 1. The process for operators to determine the level of network security protection is as follows: determine the object of the rating—preliminarily determine the level—expert review—approval by the competent authority—record review.

**Table 1:** Network security level classification standard.

Object of infringement	The degree of infringement on the object		
	General damage	Serious damage	Particularly severe damage
The legal rights and interests of citizens, legal persons and other organizations	The first level	The second level	The second level
Social order and public interests	The second level	The third level	The fourth level
National security	The third level	The fourth level	The fifth level

Different levels of communication networks have different requirements for network architecture, communication transmission and trusted authentication. When evaluating the security level of a communication network, the number of evaluation items for each index of the network of different levels is different. The specific number is shown in Table 2. For the fifth-level network, it is only necessary to judge whether the network will cause particularly severe damage to national security when the network is infringed. So, it is not shown in the table. The details of each evaluation item in Table 2 can be found in^16^.

**Table 2:** Number of evaluation items for different levels of network.

Evaluation index	Level 1	Level 2	Level 3	Level 4
Network architecture	0	2	5	6
Communication transmission	1	1	2	4
Trusted authentication	1	1	1	1

According to the security level of the communication network and the degree that each level of network meets the requirements of the national network security level evaluation, we can give the security value of the network:

5 $$ S = \left\{ {\begin{array}{*{20}l} {0,} \hfill & {Does\;{ }not{ }\;meet{ }\;the{ }\;standards} \hfill \\ {\frac{{\mathop \sum \nolimits_{i = 1}^{3} \frac{{num_{i\_m}^{item} }}{{num_{i}^{item} }}}}{3},} \hfill & {Basically{ }\;meet\;{ }the{ }\;standards} \hfill \\ \end{array} } \right. $$

In the above equation, “Does not meet the standards” refers to the existence of non-compliance items in the evaluation results of the network at this level, and the security problems in the network will cause the network to face a higher level of risk. “Basically meet the standards” means that there are items in the evaluation results that do not fully meet the standard, but these issues do not result in high risk to the network. ${num}_{i\_m}^{item}$ represents the number of evaluation items that meet the standard in the *i*_*th*_ index. ${num}_{i}^{item}$ represents the total number of evaluation items of the *i*_*th*_ index. When all the evaluation indexes of the communication network meet the requirements, *S* is 1. The degree to which the various indexes of a network meet the evaluation requirements can be evaluated by professional evaluation institutions.

The reliability value of the network is mainly related to the infrastructure reliability, the network connectivity and the data retransmission rate. The infrastructure reliability ${R}_{f}$, network connectivity ${R}_{c}$, and data retransmission rate ${R}_{t}$ are defined as follows:

6 $${R}_{f}=\left\{\begin{array}{c}\begin{array}{cc}0,& {R}_{fa}<99.999\%\end{array}\\ \begin{array}{cc}1,& {R}_{fa}\ge 99.999\%\end{array}\end{array}\right.$$

7 $${R}_{c}=\frac{{num}_{pair}}{{num}_{max}({num}_{max}-1)}$$

8 $${R}_{t}=\frac{{T}_{re}}{{T}_{total}}$$

In the above formula, ${R}_{fa}$ represents network architecture reliability, ${num}_{pair}$ represents the number of interconnected node pairs,${num}_{max}$ represents the maximum possible number of node pairs in the network, ${T}_{re}$ represents the amount of data that needs to be retransmitted, ${T}_{total}$ represents the total amount of data transmitted in the network. Since operators require network infrastructure reliability to reach more than 99.999%, ${R}_{f}$ equal to 1 becomes the premise of network reliability value. In addition, network reliability is positively correlated with network connectivity and negatively correlated with data retransmission rate. We assume that network connectivity and data retransmission rate have the same degree of impact on network reliability. This is reflected in the formula for the same absolute value of the derivative. Moreover, the reliability value of network should range from 0 to 1. For the above reasons, the reliability value $R$ can be defined as:

9 $$R={R}_{f}\frac{2{R}_{c}(1-{R}_{t})}{1+{R}_{c}-{R}_{t}}$$

The partial derivative of the above formula can be obtained:

10 $$\frac{\partial R}{\partial {R}_{c}}={R}_{f}\frac{2{{R}_{t}}^{2}}{{(1+{R}_{c}-{R}_{t})}^{2}}$$

11 $$\frac{\partial R}{\partial {R}_{t}}=-{R}_{f}\frac{2{{R}_{c}}^{2}}{{(1+{R}_{c}-{R}_{t})}^{2}}$$

It can be seen from the above two equations that the absolute values of the two partial derivative are equal. This verifies the assumption that network connectivity and data retransmission rate have the same effect on network reliability. $\frac{\partial R}{\partial {R}_{c}}$ is a positive number indicating that network reliability is positively correlated with network connectivity. $\frac{\partial R}{\partial {R}_{t}}$ is a negative number indicating that network reliability is negatively correlated with the data retransmission rate.

The final safe and reliable value model is as follows:

12 $${V}_{safe}=S\times R=S\times {R}_{f}\times \frac{2{R}_{c}(1-{R}_{t})}{1+{R}_{c}-{R}_{t}}$$

It can be seen from the above formula that when the network does not meet the requirements of the security level at all or the architecture reliability of the network does not reach 99.999%, the safe and reliable value of the network is directly equal to zero. Therefore, the premise that a network has safe and reliable value is that the network is basically secure and the network architecture is reliable.

### Quantitative model of QoS value

From the second section, we can know that the QoS value of the network is related to four indicators: bandwidth satisfaction, packet loss satisfaction, delay satisfaction and delay jitter satisfaction. The weighted sum of these four indicators is used to construct the QoS value model of the network. It is defined as follows:

13 $${V}_{QoS}=\sum {W}_{i}{Q}_{i}$$

where ${W}_{i}$ represents the weight of each index, ${Q}_{i}$ represents the value of each index.

Here, the weight refers to the quantitative contribution of each indicator to QoS value. Analytic Hierarchy Process (AHP) is a simple, flexible and practical method to quantitatively determine the weight of indicators^18^. In this paper, AHP is applied to determine the weight of each index. AHP generally decomposes the problem into three levels, namely the target level, the criterion level and the scheme level. Based on the judgment of objective reality, this method determines the relative importance of each factor in each level through pairwise comparison, and expresses it quantitatively. Then the weight of relative importance of all factors at each level is determined by mathematical method to provide reference for the final decision. In this paper, we only need to calculate the relative importance weights of all indicators in QoS, so only hierarchical single sorting is required. The relative importance between each index is determined by Delphi Method^19^. Next, the weight of each index is calculated.

First, the expert’s indices comparison matrix is constructed by Delphi Method. We invited 20 professors, researchers and other experts to independently compare QoS value indicators pair by pair^20^. And they were asked to rate the importance of each factors using nine-point scale. The meaning of different scores is shown in Table 3.

**Table 3:** Pair-wise comparison scale for AHP importance.

Numerical rating	Verbal judgments of importance
1	Equal importance
3	Moderate importance
5	Strong importance
7	Very strong importance
9	Extreme importance
2, 4, 6, 8	Intermediate value of adjacent judgment
Reciprocal	The importance of the two indicators is contrary to the above results

According to the scoring results of experts, a comparison matrix can be constructed. Here, one of the results is taken as an example for analysis. The scoring results are shown in Table 4. The “3” in the first column of Table 4 indicates that the delay satisfaction is moderately more important than the bandwidth satisfaction, and the last “1/3” in the second column indicates that the packet loss satisfaction is moderately important than the delay jitter satisfaction.

**Table 4:** Pair-wise comparison results.

	${Q}_{bs}$	${Q}_{pl}$	${Q}_{d}$	${Q}_{j}$
${Q}_{bs}$	1	$1/5$	$1/3$	$1/2$
${Q}_{pl}$	5	1	3	3
${Q}_{d}$	3	$1/3$	1	1
${Q}_{j}$	2	$1/3$	1	1

According to the above pair-wise comparison results, a comparison matrix can be constructed:

14 $$Q=\left[\begin{array}{cccc}1& 1/5& 1/3& 1/2\\ 5& 1& 3& 3\\ 3& 1/3& 1& 1\\ 2& 1/3& 1& 1\end{array}\right]$$

The eigenvalue of matrix $Q$ can be calculated by the following formulas:

15 $$Qx=\lambda x\Rightarrow (Q-\lambda E)x=0\Rightarrow |Q-\lambda E|=0$$

16 $$|Q-\lambda E|=\left|\begin{array}{cccc}1-\lambda & 1/5& 1/3& 1/2\\ 5& 1-\lambda & 3& 3\\ 3& 1/3& 1-\lambda & 1\\ 2& 1/3& 3& 1-\lambda \end{array}\right|=0$$

Solving the above formula, the maximum eigenvalue is ${\lambda }_{max}=4.0335$. The corresponding eigenvector is ${[0.1454 0.8776 0.3405 0.3045]}^{T}$.

To prevent the results of the hierarchical single sort from being too random, it is necessary to judge whether the current result meets the consistency test standard. The Consistency Index ($CI$) can be calculated by the maximum eigenvalue ${\lambda }_{max}$, as shown in Eq. (17).

17 $$CI=\frac{{\lambda }_{max}-n}{n-1}$$

where $n$ is the size of matrix.

Next, it is necessary to calculate the Consistency Ratio ($CR$). The definition formula is shown in Eq. (18). If $CR$ is greater than 0.1, it indicates that the current judgment matrix is highly random and does not meet the consistency test. Therefore, appropriate modifications are required. Only when $CR$ is less than 0.1, the result is valid.

18 $$CR=CI/RI$$

In the above equation, $RI$ is the average random consistency index, which can be obtained from Table 5.

**Table 5:** Average random consistency index ($RI$)^21^.

*n*	1	2	3	4	5	6	7	8	9	10
*RI*	0	0	0.58	0.9	1.12	1.24	1.32	1.41	1.45	1.49

In this paper, there are four QoS value indicators, so $n$ is equal to 4 and $RI$ is 0.9. Therefore, the calculation results of $CI$ and $CR$ are as follows:

19 $$CI=\frac{{\lambda }_{max}-n}{n-1} =\frac{4.0335-4}{4-1}=0.0112$$

20 $$CR=\frac{CI}{RI}=\frac{0.0112}{0.9}=0.0124<0.1$$

It can be seen from the above formula that the obtained result meets the consistency test. So, the result is valid. Finally, the weight ${W}_{sample}$ can be obtained by normalizing the eigenvector corresponding to the maximum eigenvalue.

21 $${W}_{sample}=\frac{{W}_{i}}{{\sum }_{i=1}^{4}{W}_{i}}={\left[\begin{array}{cccc}0.0872& 0.5261& 0.2042& 0.1825\end{array}\right]}^{T}$$

The processing procedures for other samples are the same as the above sample. Finally, take the average weight of all samples to get the final weight:

22 $$W=\frac{{W}_{{sample}_{i}}}{{\sum }_{i=1}^{20}{W}_{{sample}_{i}}}={\left[\begin{array}{cccc}0.2684& 0.4750& 0.1546& 0.1020\end{array}\right]}^{T}$$

Therefore, the constructed QoS value model is as follows:

23 $${V}_{QoS}=\sum {W}_{i}{Q}_{i}=0.2684{Q}_{bs}+0.4750{Q}_{pl}+0.1546{Q}_{d}+0.1020{Q}_{j}$$

### Quantitative model of economic value

The solution to the economic value can be decomposed into two parts: revenue and cost. Network revenue is mainly related to the network resources, the unit price of resources, and the number of users. Assuming that the annual revenue of the communication network is constant under the condition that the total network resources remain unchanged, the network revenue is defined as follows:

24 $${R}_{Net}=Y[{\sum }_{j=0}^{N}{\sum }_{i=0}^{m}{x}_{ij}{\alpha }_{1}{y}_{ij}+{\sum }_{j=0}^{N}{\sum }_{i=0}^{n}{x}_{ij}{\alpha }_{2}{y}_{ij}]$$

where ${\alpha }_{1}$ and ${\alpha }_{2}$ are represent the unit price of bandwidth for household users and the unit price of bandwidth for enterprise users, respectively. ${y}_{ij}$ represents the amount of resources allocated to user *i* by link *j*. ${x}_{ij}$ can only be 0 or 1. The value of 0 indicates that user *i* is not connected to link *j*, and the value of 1 indicates that there is a connection. *N* represents the number of links, *m* represents the number of household users, and *n* represents the number of enterprise users. *Y* represents the year of network usage. As the years of network usage increase, network revenues gradually increase.

The cost of a communication network mainly includes capital expenditures (CAPEX) and operational expenditures (OPEX). CAPEX are accounted for the first year^15^. It can be expresses by:

25 $${C}_{capex}={C}_{fd}+{C}_{eq}$$

where ${C}_{fd}$ and ${C}_{eq}$ are the optical-fiber deployment cost and the equipment cost, respectively. OPEX can be decomposed as:

26 $${C}_{opex}={C}_{co}+m{C}_{O\&M\_H}+n{C}_{O\&M\_E}$$

where ${C}_{co}$, ${C}_{O\&M\_H}$ and ${C}_{O\&M\_E}$ are the operation and maintenance ($O\&M$) cost of the central office, the household user end and the enterprise user end, respectively.

Assuming that OPEX is increasing year by year, the increase amount is ${C}_{opex\_incre}$. The total cost of using the network for *Y* years is:

27 $$C={C}_{capex}+Y \, {C}_{opex}+\frac{Y\left(Y-1\right)}{2}{C}_{opex\_incre}={C}_{fd}+{C}_{eq}+Y\left({C}_{co}+m{C}_{O\&M\_H}+n{C}_{O\&M\_E}\right)+\frac{Y\left(Y-1\right)}{2}{C}_{opex\_incre}$$

The economic value of network is defined as:

28 $$ V_{E} = R_{Net} - C = Y\left[ {\mathop \sum \limits_{j = 0}^{N} \mathop \sum \limits_{i = 0}^{m} x_{ij} \alpha_{1} y_{ij} + \mathop \sum \limits_{j = 0}^{N} \mathop \sum \limits_{i = 0}^{n} x_{ij} \alpha_{2} y_{ij} } \right] - \left[ {C_{fd} + C_{eq} + Y\left( {C_{co} + mC_{O\& M\_H} + nC_{O\& M\_E} } \right) + \frac{{Y\left( {Y - 1} \right)}}{2}C_{opex\_incre} } \right] $$

The normalization of network economic value can be calculated by the ratio of the current value to the maximum value:

29 $${V}_{E}=\frac{{V}_{E}}{{V}_{Emax}}$$

30 $${V}_{Emax}={R}_{Net\_max}-{C}_{min}$$

31 $${R}_{Net\_max}={\sum }_{j=0}^{N}{y}_{j}\cdot \mathrm{max}\{{\alpha }_{1},{\alpha }_{2}\}$$

32 $${C}_{min}={C}_{capex}+Y{C}_{opex\_min}+\frac{Y\left(Y-1\right)}{2}{C}_{opex\_incre}$$

### Multi-dimensional quantitative model of network value

In the previous subsections, the values of the three dimensions were modeled. In this section, the communication network value will be modeled. The communication network value is the weighted sum of each dimension value. Similarly, the hierarchical single sorting method is used to determine the weights of different dimensions. The judgment matrix obtained is as follows:

33 $$V=\left[\begin{array}{ccc}1& 2& 2\\ 1/2& 1& 2\\ 1/2& 1/2& 1\end{array}\right]$$

The maximum eigenvalue of the matrix *V* is ${\lambda }_{max}=3.0536$. The corresponding eigenvector is ${[0.8021 0.5053 0.3183]}^{T}$.

34 $$CI=\frac{{\lambda }_{max}-n}{n-1} =\frac{3.0536-3}{3-1}=0.0268$$

35 $$CR=\frac{CI}{RI}=\frac{0.0268}{0.58}=0.0462<0.1$$

As can be seen from the above equation, the solution conforms to the consistency test. The weight *W* of each dimension value can be gotten by normalizing the eigenvector corresponding to the maximum eigenvalue:

36 $$W=\frac{{W}_{i}}{{\sum }_{i=1}^{3}{W}_{i}}={\left[\begin{array}{ccc}0.4934& 0.3108& 0.1958\end{array}\right]}^{T}$$

The final quantitative model of network value is as follows:

37 $$ \begin{aligned} V & = 0.4934V_{safe} + 0.3108V_{QoS} + 0.1958V_{E} \\ & = 0.4934 \times S \times R_{f} \times \frac{{2R_{c} \left( {1 - R_{t} } \right)}}{{1 + R_{c} - R_{t} }} \\ & \;\;\;\; + \;0.3108\left[ {0.2684Q_{bs} + 0.4750Q_{pl} + 0.1546Q_{d} + 0.1020Q_{j} } \right] \\ & \;\;\;\; + \;0.1958 \times \frac{{R_{Net} - C}}{{R_{Net\_max} - C_{min} }} \\ \end{aligned} $$

## Network value analyses

**Figure 2:**
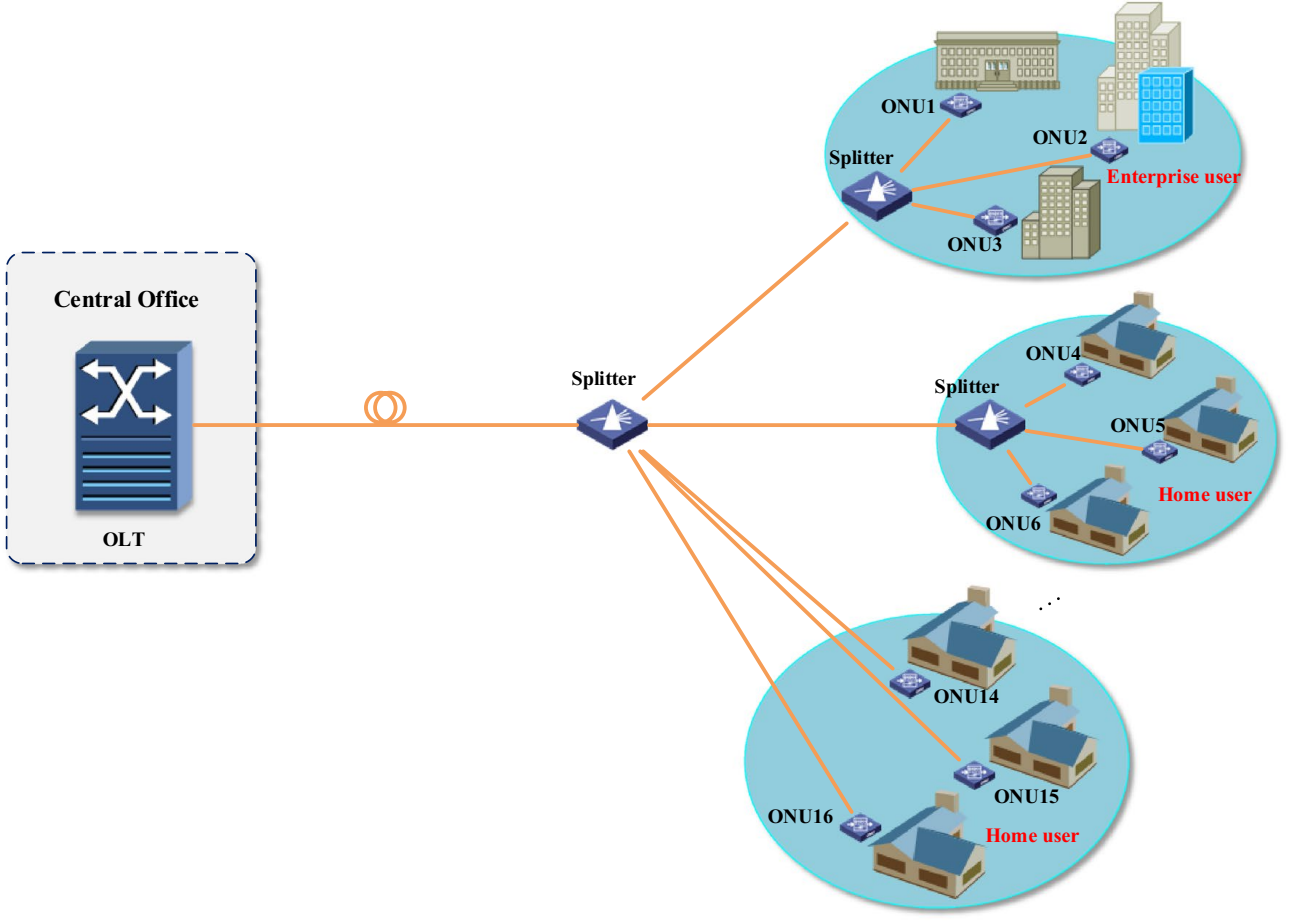
EPON architecture.

The model proposed in this paper is applicable to all optical communication networks. To show how the model can be applied to optical networks, in this section, we analyze the network value of an EPON. The EPON architecture used in this paper is shown in Fig. 2. The network consists of one optical line terminal (OLT) and 16 optical network units (ONUs). Three ONUs serve enterprise users and 13 ONUs serve household users. The network link transmission rate is 1 Gb/s. At the OLT end, Interleaved polling with adaptive cycle time (IPACT) and Cyclic polling with fixed cycle time (CPFCT) algorithms are used for DBA^22,23^. In order to be suitable for the communication network adopted in this paper, IPACT and CPFCT algorithms are adjusted to ensure QoS performance of users with high priority. The safe and reliable value, QoS value, economic value and overall value of the network are respectively analyzed in the following.

### Analysis of safe and reliable value

When the EPON network used in this paper is damaged, it will cause serious damage to individuals or organizations. So, the network is a third-level security protection network. Assume that the network meets all the evaluation requirements of the third-level security network, and the infrastructure reliability meets the requirement of 99.999%, that is, $S=1$, ${R}_{f} =1$. Because all ONUs are connected to OLT in this network, the network connectivity is 1.

**Figure 3:**
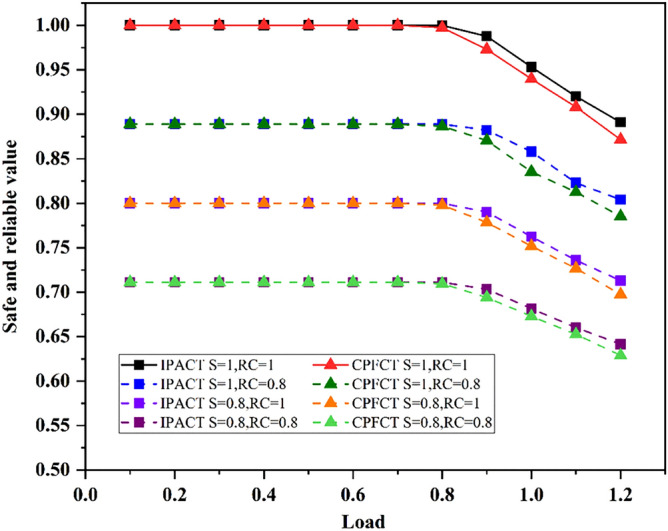
Safe and reliable value.

The safe and reliable values of network under different security scenarios are shown in Fig. 3. As can be seen from the solid line in the figure, when the network load is less than 0.8, the safe and reliable values of the two algorithms are almost the same. This is because when load is light, no matter what algorithm is adopted, the data retransmission rate of the network can always keep very low. When the load exceeds 0.8, the safe and reliable value of network begins to decline. This is because the data packet processing rate is lower than the data packet generation rate at this time. And the data packet is prone to transmission failure, resulting in a higher data retransmission rate and lowering the safe and reliable value. With the decrease of network security value or network connection rate, the safe and reliable value of network is greatly reduced. Moreover, the impact of reduced security value on the safe and reliable value of network is greater than the impact of reduced network connection rates. It also shows that security value has a greater weight. As can be seen from Fig. 3, network security, network connectivity and the use of different DBA algorithms will affect the safe and reliable value of the network. Operators can improve the safe and reliable value of the network through these three aspects.

### Analysis of QoS value

**Figure 4:**
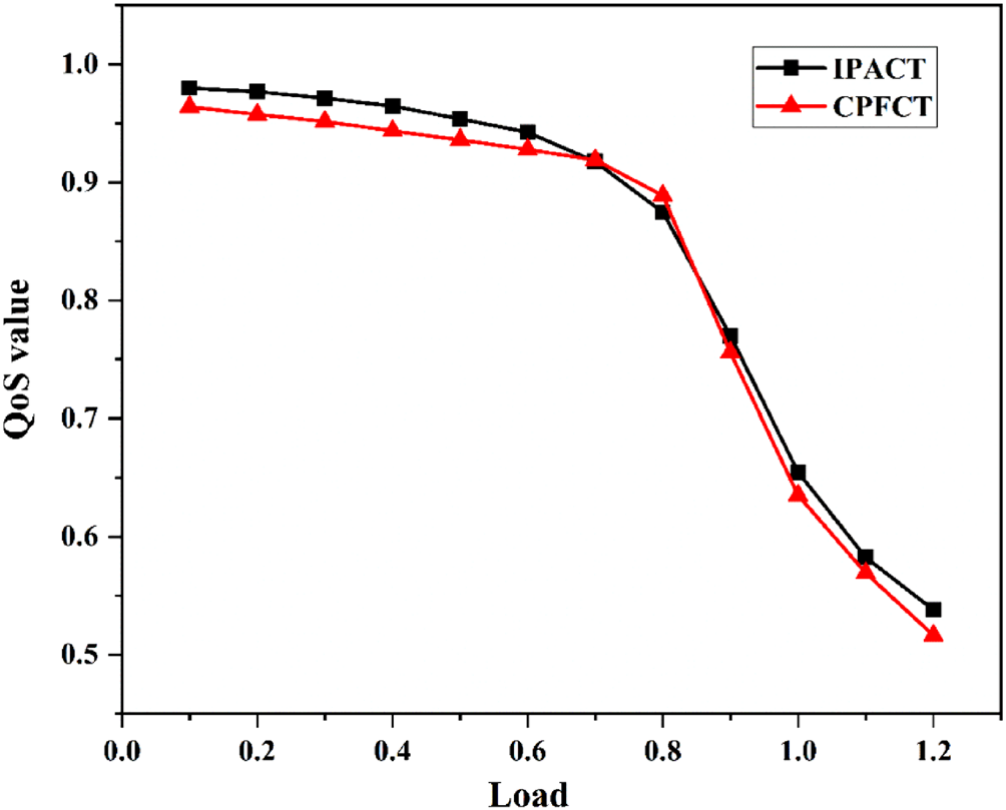
Network QoS value of different algorithms.

This section analyzes the QoS value of the network. The values of $\delta$ in formula (1) and formula (2) are 1 and 5 respectively^13^. The value of ${d}_{m}$ in formula (3) is half of the average delay when the load is 0.9, and $\delta$ is 1. The value of ${d}_{jm}$ in formula (4) is half of the delay jitter when the load is 0.9, and $\delta$ is 1. When using different DBA algorithms, the QoS value curve of the EPON is shown in Fig. 4. It can be seen from the figure that when the network load is less than 0.7, the network QoS value is greater than 0.9. Because the bandwidth, delay, delay jitter, and packet loss rate of the network can meet user needs at this time. When the load is greater than 0.7, as the load increases, the QoS value of the network begins to drop sharply. This is because when the network load is heavy, the resources of the network are not enough to meet the needs of all users, which leads to a decrease in user satisfaction. On the whole, the QoS value of IPACT algorithm is greater than that of CPFCT algorithm. This indicates that different algorithms have different effects on network QoS value.

**Figure 5:**
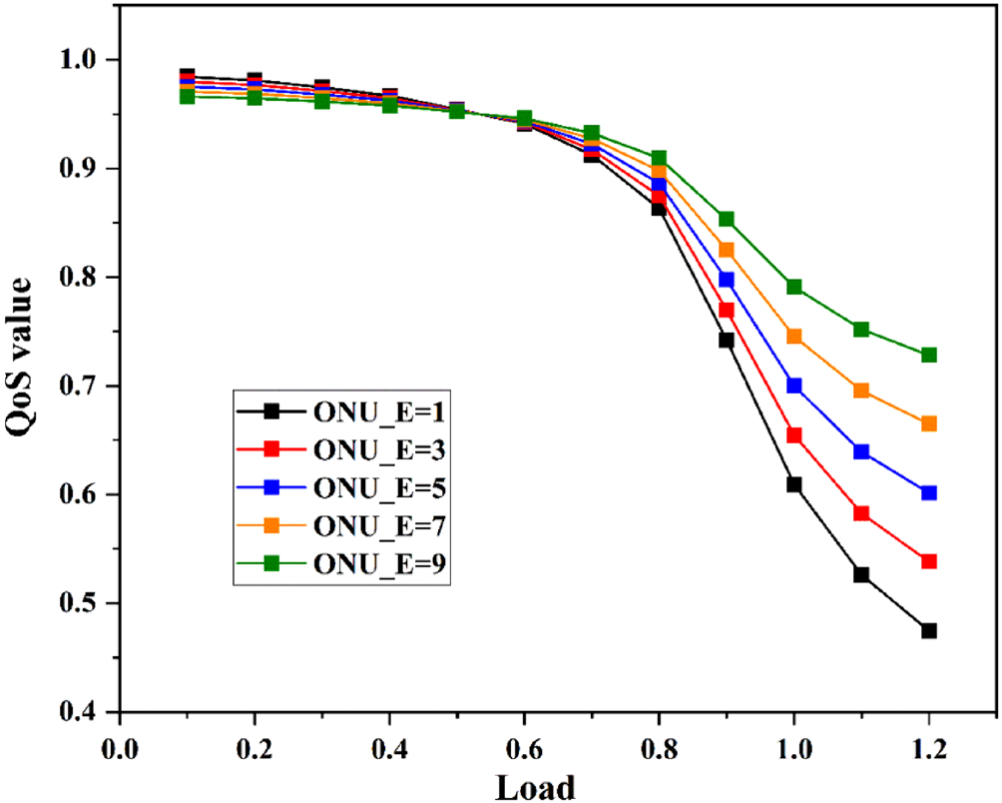
Network QoS value of different enterprise users.

When IPACT algorithm is adopted, the QoS value of network changes with the number of ONUs serving enterprise users, as shown in Fig. 5. While the load is less than 0.7, the QoS value of networks with different numbers of enterprise users has little difference. When the load is greater than 0.7, with the increase of the number of enterprise users in the network, the QoS value of the network gradually increases. And the greater the load, the greater the increase. This is because enterprise users have a higher priority. When under heavy load, the QoS of enterprise users can still be guaranteed. Correspondingly, the QoS value of the network will increase.

### Analysis of economic value

**Figure 6:**
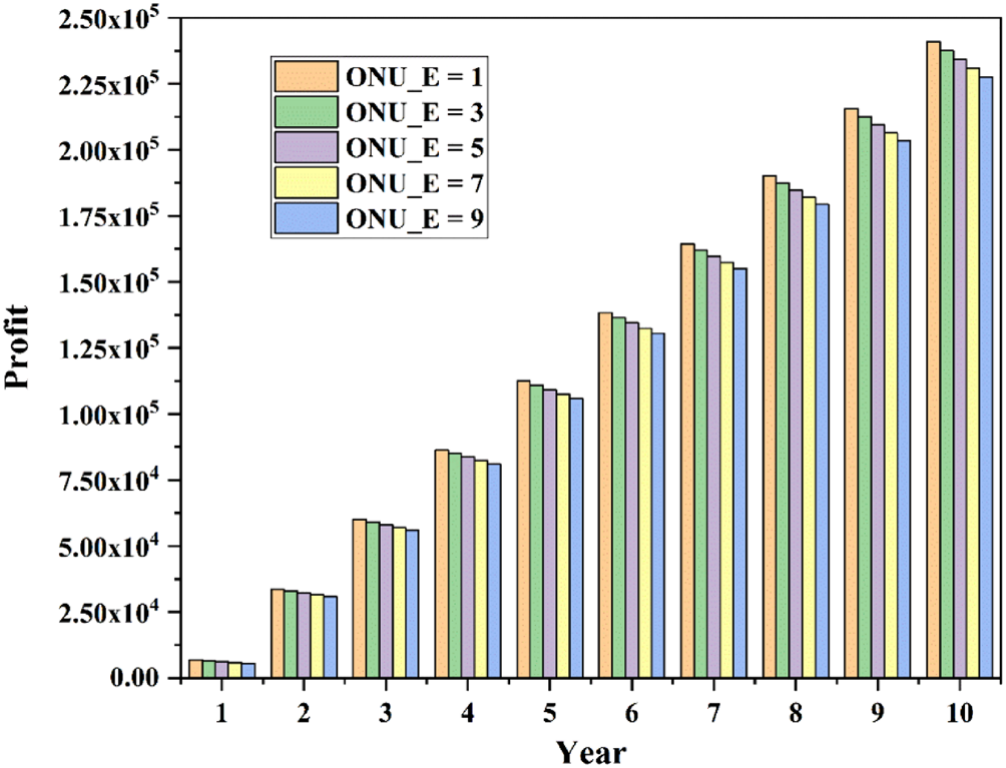
EPON profit varies with year.

The economic value of the network is analyzed in the following part. The values of each parameter in formulas (24)–(32) are shown in Table 6. Assume that up to 25 household users can share 100Mbps. The change of EPON network profit over the years is shown in Fig. 6. It can be seen from the figure that in the first year, the network generated very little profit. This is because of the high cost of network construction. With the year increase, network profits will gradually increase. However, as the number of enterprise users increases, network profits show a downward trend. And the greater the number of years, the greater the reduction. This is because when total network capacity is constant, the number of household users that the network can accommodate is much more than that of enterprise users. Although the unit price of bandwidth for enterprise users is much higher than that for home users, the network’s profit through home users is still greater than that for enterprise users.

**Table 6:** Parameters and corresponding values^15,24,25^.

Parameters	Costs ($)
Fiber	100/km
OLT	2000
ONU	75/unit
Splitter	2.5/port
Optical-fiber deployment cost ($${C}_{fd}$$)	800
Central office computer room construction cost	15,000
Central office annual O&M ($${C}_{co}$$)	1200
Enterprise users annual O&M ($${C}_{O\&M\_E}$$)	55/unit
Household users annual O&M ($${C}_{O\&M\_H}$$)	7.5/unit
Annual O&M increment ($${C}_{opex\_incre}$$)	150
Unit price of bandwidth for household users ($${\alpha }_{1}$$)	1.2/Mbps/year
Unit price of bandwidth for enterprise users ($${\alpha }_{2}$$)	27/Mbps/year

**Figure 7:**
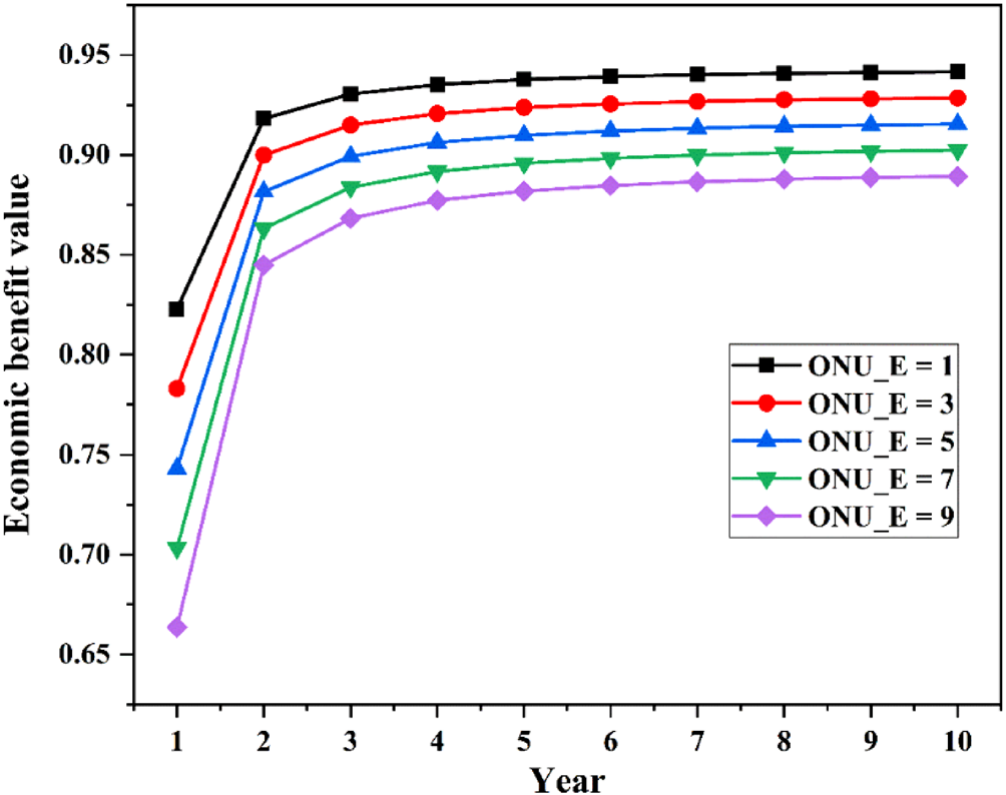
Economic value.

The curve of network economic value over the years is shown in Fig. 7. It can be seen from the figure that the economic value of the network in the first year is relatively low. Starting from the second year, with the increase of the network service life, the economic value of network gradually increases, and finally tends to be stable. As the number of enterprise users increases, the value of network economic benefits declines, which is consistent with changes in network profits. In general, the value of economic benefits can be improved by reducing network costs, or by reasonably planning the number of enterprise users and household users.

### Analysis of network value

**Figure 8:**
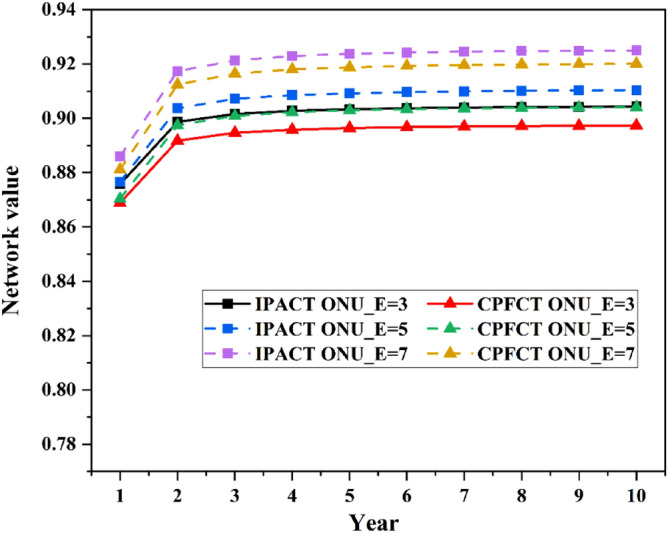
Network value varies with the number of enterprise users.

The first three subsections analyzed the value of each dimension respectively, and this part analyzes the overall value of the network. Both the safe and reliable value and QoS value are taken the value when load is 0.9. The network values change with the number of enterprise users are shown in Fig. 8. Among them, the solid line is the network value of EPON used in this article, the dotted line is the network value when changing the number of enterprise users. As can be seen from the figure, the network value in the first year is much lower than that in other years. This is because the economic value of network in the first year is very low. From the second year on, the network value changed little with the year and basically maintained at a stable value. As the number of enterprise users increases, the value of the network increases. This is because QoS value of the network increases with the number of enterprise users. Although the economic value of the network decreases with the increase of the number of enterprise users, the increase of QoS value makes up for the decrease of the economic value. So, the value of the network increases. In real life, the number of users of the network will continue to change, and the algorithms used by the network will also be updated. Therefore, in practical applications, the network value continues to change even after the second year.

**Figure 9:**
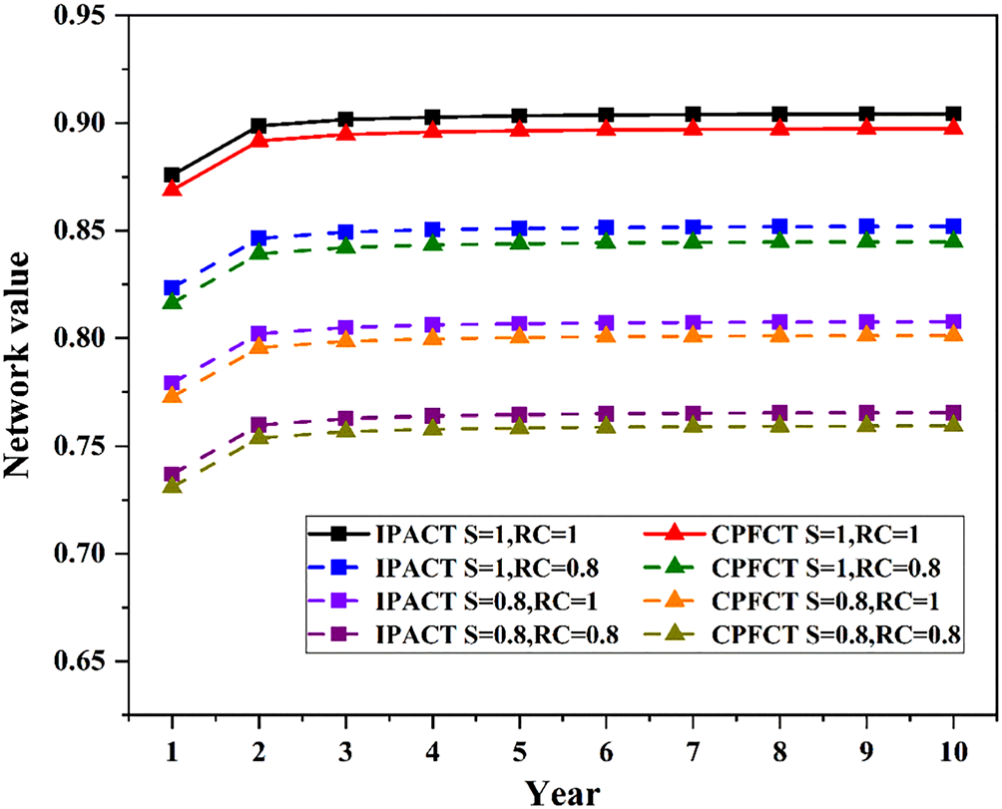
Network value in different security scenarios.

The network values in different security scenarios are shown in Fig. 9. The figure shows that when security value of network is reduced, the network value will be greatly reduced. Similarly, when the network connectivity decreases, the network value will also decrease. And the impact of security value reduction on network value is much greater than network connectivity. Compared with Fig. 8, the curve in Fig. 9 has a larger change. This is determined by the weight of indicator. The safe and reliable value occupies the largest weight in the network value, so it has the greatest influence on the network. At the same time, it can be seen from the two figures that different algorithms have different effects on network value. Comparatively, the network using IPACT algorithm has higher network value than using CPFCT algorithm.

Like the single OLT network mentioned above, the value evaluation of a multi-OLT network is also carried out from three dimensions: safe and reliable value, QoS value and economic value. For the safe and reliable value, the security of multi-OLT networks is evaluated in the same way as the security of single OLT networks. In terms of reliability, the connection rate of a multi-OLT network should consider the connection between the ONU and all OLTs. For QoS value and economic value, there is no difference between the quantitative model of multi-OLT network and single-OLT network. It is just that the algorithm adopted by the multi-OLT network needs to consider the resource allocation among the multiple OLTs.

In general, there are three ways to improve the network value. The first is to make the network indicators meet the corresponding level evaluation requirements as much as possible to improve the security value of the network. The second is that the network value can be enhanced by rationally planning the number of enterprise users and the number of home users. Finally, higher performance DBA algorithms can be used to enhance the value of the network.

## Conclusion

We constructed a multi-dimensional quantitative model of communication network value for the first time, and quantified the abstract concept of network value. At first, we analyzed various factors that influence the evaluation of network value from multiple perspectives, and established an index system of multi-dimensional network value evaluation. Next, the AHP method was adopted to determine the weight of each dimension and the weight of different indicators in the second dimension. Furthermore, according to the characteristics of each dimension and its weight in the evaluation system, a multi-dimensional quantitative model of communication network value was constructed. Finally, the value of EPON was calculated and analyzed in detail, and three strategies were proposed for operators to increase the network value.

## Data Availability

The code and relevant dataset have been published in the following URL: https://github.com/Echoooooo121/network-value.git.
